# Testing a very low-carbohydrate adaption of the Diabetes Prevention Program among adults with prediabetes: study protocol for the Lifestyle Education about prediabetes (LEAP) trial

**DOI:** 10.1186/s13063-022-06770-3

**Published:** 2022-09-30

**Authors:** Dina H. Griauzde, Alison O’Brien, William S. Yancy, Caroline R. Richardson, Jamie Krinock, Melissa DeJonckheere, Deanna J. M. Isaman, Kaitlyn Vanias, Samuel Shopinski, Laura R. Saslow

**Affiliations:** 1 Department of Internal Medicine, University of Michigan Medical School, 2800 Plymouth Road, Building 16, Room 16-371C, Ann Arbor, MI 48109-2800 USA; 2 VA Ann Arbor Healthcare System, Ann Arbor, MI USA; 3 University of Michigan Institute for Healthcare Policy and Innovation, Ann Arbor, MI USA; 4 Department of Health Behavior and Biological Sciences, University of Michigan School of Nursing, Ann Arbor, MI USA; 5 Department of Medicine, Duke University School of Medicine, Durham, NC USA; 6 Department of Family Medicine, University of Michigan Medical School, Ann Arbor, MI USA; 7 University of Wisconsin Hospitals and Clinics, Madison, WI USA; 8 National Kidney Foundation of Michigan, Ann Arbor, MI USA

**Keywords:** Prediabetes, Type 2 diabetes, Prevention, Diabetes Prevention Program, Dietary carbohydrate restriction, Very low-carbohydrate diet

## Abstract

**Background:**

The Center for Disease Control and Prevention’s National Diabetes Prevention Program (NDPP) aims to help individuals with prediabetes avoid progression to type 2 diabetes mellitus (T2DM) through weight loss. Specifically, the NDPP teaches individuals to follow a low-fat, calorie-restricted diet and to engage in regular physical activity to achieve ≥ 5% body weight loss. Most NDPP participants, however, do not achieve this weight loss goal, and glycemic control remains largely unchanged. One promising opportunity to augment the NDPP’s weight loss and glycemic effectiveness may be to teach participants to follow a very low-carbohydrate diet (VLCD), which can directly reduce post-prandial glycemia and facilitate weight loss by reducing circulating insulin and enabling lipolysis. To date, there have been no high-quality, randomized controlled trials to test whether a VLCD can prevent progression to T2DM among individuals with prediabetes. The aim of this study is to test the effectiveness of a VLCD version the NDPP (VLC-NDPP) versus the standard NDPP. We hypothesize the VLC-NDPP will demonstrate greater improvements in weight loss and glycemic control.

**Methods:**

We propose to conduct a 12-month, 1:1, randomized controlled trial that will assign 300 adults with overweight or obesity and prediabetes to either the NDPP or VLC-NDPP. The primary outcome will be glycemic control as measured by change in hemoglobin A1c (HbA1c) from baseline to 12 months. Secondary outcomes will include percent body weight change and changes in glycemic variability, inflammatory markers, lipids, and interim HbA1c. We will evaluate progression to T2DM and initiation of anti-hyperglycemic agents. We will conduct qualitative interviews among a purposive sample of participants to explore barriers to and facilitators of dietary adherence. The principal quantitative analysis will be intent-to-treat using hierarchical linear mixed effects models to assess differences over time.

**Discussion:**

The NDPP is the dominant public health strategy for T2DM prevention. Changing the program’s dietary advice to include a carbohydrate-restricted eating pattern as an alternative option may enhance the program’s effectiveness. If the VLC-NDPP shows promise, this trial would be a precursor to a multi-site trial with incident T2DM as the primary outcome.

**Trial registration:**

NCT05235425. Registered February 11, 2022.

## Administrative information

Note: the numbers in curly brackets in this protocol refer to SPIRIT checklist item numbers. The order of the items has been modified to group similar items (see http://www.equator-network.org/reporting-guidelines/spirit-2013-statement-defining-standard-protocol-items-for-clinical-trials/).

**Table:** 

Title {1}	Testing a very low-carbohydrate adaption of the Diabetes Prevention Program among adults with prediabetes: study protocol for the Lifestyle Education about Prediabetes (LEAP) trial
Trial registration {2a and 2b}.	NCT05235425. Clinicaltrials.gov
Protocol version {3}	Version 1.0 HUM0019546 Approved March 21, 2022
Funding {4}	Funding to support this work was provided by the National Institutes of Health, R01DK125792. Trial conducted in partnership with the National Kidney Foundation of Michigan (NKFM).
Author details {5a}	Department of Internal Medicine, University of Michigan Medical School, Ann Arbor, MI VA Ann Arbor Healthcare System, Ann Arbor, MI University of Michigan Institute for Healthcare Policy and Innovation, Ann Arbor, MI Department of Health Behavior and Biological Sciences, University of Michigan School of Nursing, Ann Arbor, MI Department of Medicine, Duke University School of Medicine, Durham, NC Department of Family Medicine, University of Michigan Medical School, Ann Arbor, MI University of Wisconsin Hospitals and Clinics, Madison, WI National Kidney Foundation of Michigan, Ann Arbor, MI
Name and contact information for the trial sponsor {5b}	National Institute of Diabetes and Digestive and Kidney Diseases (NIDDK) 9000 Rockville Pike Bethesda, MD 20,892
Role of sponsor {5c}	The funder will have no role in data collection and analysis, decision to publish, or preparation of the manuscript.

## Introduction

### Background and rationale {6a}

In the USA, type 2 diabetes mellitus (T2DM) is a leading cause of morbidity, mortality, and health care spending with total estimated costs of $327 billion in 2017 [1, 2]. T2DM is also preventable; the landmark Diabetes Prevention Program (DPP) trial demonstrated that a resource-intensive, one-on-one program combining diet and physical activity can reduce the 3-year risk of T2DM by 58% compared with usual care [3]. The DPP intensive lifestyle intervention taught participants to follow a low-fat, calorie-restricted diet and encouraged individuals to engage in at least 150 min of moderate-intensity physical activity per week.

The Centers for Disease Control and Prevention (CDC) adapted the diet and physical activity recommendations provided in the original DPP trial into a group-based program, which is now offered at over 1,500 sites throughout the USA and reimbursed by Medicare and other payors [4, 5]. For quality assurance, the CDC oversees a Diabetes Prevention Recognition Program (DPRP), which requires organizations to meet specific standards, including use of a CDC-approved curriculum [6]. The CDC offers two approved curricula for public use: (1) 2012 National Diabetes Prevention Program (NDPP) or (2) *PreventT2*. Consistent with the original DPP trial, the 2012 NDPP emphasizes dietary fat restriction through adherence to explicit daily fat gram limits. In contrast, *PreventT2* does not provide an explicit daily fat gram limit, but rather generally encourages participants to limit fat, calories, and sugar. To date, no studies have evaluated the comparative effectiveness of the 2012 NDPP to *PreventT2*, and most studies of real-world DPPs have used the 2012 NDPP [7].

Despite the widespread availability of community-based DPPs and CDC quality assurance efforts [6], DPP attrition is high (~ 50% at 6 months) and most participants (64%) do not achieve goal weight loss of ≥ 5% [7, 8]. Hemoglobin A1c (HbA1c) is not routinely measured in real-world DPPs, as weight loss is the key driver of T2DM risk reduction [9]. Among the few DPP effectiveness studies that evaluated glycemic change, the average reduction in HbA1c was 0.07% [10]. These data suggest the need for new strategies to augment the real-world effective of DPPs and reduce the individual and public health consequences of prediabetes and T2DM.

One key opportunity to support weight loss and glycemic improvements among DPP participants may be by modifying the program’s dietary advice. The DPP lifestyle intervention was developed over 30 years ago, when dietary fat restriction was the dominant public health recommendation to prevent and manage chronic disease. Growing data [11] and clinical practice guidelines [12] now support use of dietary carbohydrate restriction as an effective alternative strategy, particularly among patients with T2DM. Low- and very low-carbohydrate diets (respectively defined as < 130 g and < 50 g of total carbohydrate per day) [13] can reduce post-prandial glycemia, facilitate lipolysis, and weight loss through a reduction in circulating insulin [14] and minimize the need for anti-hyperglycemic medications [12, 15, 16].

Less is known, however, about the role of dietary carbohydrate restriction for actual prevention of T2DM. Consensus guidelines suggest the need for personalized nutrition therapy with a focus on self-monitoring and modifying dietary carbohydrate intake among individuals with prediabetes [11]. To explore the role of dietary carbohydrate restriction among patients with prediabetes, our team previously pilot tested a very low-carbohydrate adaptation of the CDC’s NDPP. We demonstrated that a very low-carbohydrate Diabetes Prevention Program is feasible and acceptable, with participants (*n* = 21) attending an average of 10.3/16 weekly sessions and 3.4/7 biweekly or monthly sessions [17].

### Objectives {7}

We now plan to conduct a randomized controlled trial to test the comparative effectiveness of the NDPP versus the VLC-NDPP. This study has 3 aims. First, we aim to compare change in HbA1c from baseline to 12 months. We hypothesize that the average reduction in HbA1c will be greater among VLC-NDPP participants compared to the NDPP group. Second, we aim to explore changes in other measures associated with T2DM risk, such as body weight, body fat percentage, glycemic variability, lipids, and inflammation. We hypothesize that the average changes in these measures will be more favorable in the VLC-NDPP group. While low-density lipoprotein (LDL) cholesterol may increase when following a low-carbohydrate eating pattern [18], we hypothesize that any increase in LDL cholesterol will be due to increases in larger, less atherogenic low-density lipoprotein particles [19]. Third, we aim to explore individuals’ experiences, including perceived barriers to and facilitators of dietary change and their ability to maintain these changes over time through qualitative interviews with a purposive sample of program participants.

### Trial design {8}

This is a 12-month, parallel group, randomized controlled trial. Individuals with overweight or obesity and prediabetes (n = 300) will be assigned using 1:1 randomization to one of two treatment arms: (1) NDPP or (2) VLC-NDPP. Both treatment arms will participate in a 12-month lifestyle change intervention delivered through online, group-based classes. The primary outcome will be change in glycemic control measured by HbA1c at 12 months. Secondary outcomes will include interim HbA1c change at 4 months and changes in percent body weight, percent body fat, lean body mass, glycemic variability, inflammatory markers, small particle LDL, HDL, and triglycerides at 4 and 12 months. We will also evaluate progression to T2DM and initiation of anti-hyperglycemic agents. This is a superiority trial.

## Methods

This study was approved by the University of Michigan Institutional Review Board (HUM00196546). The protocol was designed according to the Standard Protocol Items: Recommendations for Interventional Trials 2013 (SPIRIT) [20]. SPIRIT subheadings are used throughout the manuscript and noted in curly brackets. Trial registration: clinicaltrials.gov, NCT05235425. Registered February 11, 2022, https://clinicaltrials.gov/ct2/show/NCT05235425.

### Study setting {9}

The intervention will be delivered remotely by Zoom™, a HIPAA-compliant video conferencing software. All data will be collected in the USA.

### Eligibility criteria {10}

Inclusion criteria include the following: (1) aged 21–75 years; (2) overweight, defined as BMI ≥ 25 kg/m^2^ or ≥ 23 kg/m^2^ if of Asian descent; (3) HbA1c of 5.7 to 6.4% (as measured on baseline blood draw); (4) willingness and ability to participate in group-based, online sessions using audio and video; (5) ability to engage in at least light physical activities, such as walking; (6) willingness to follow either prescribed diet through random assignment; (7) willingness to self-monitor weight, dietary intake, and physical activity minutes; and (8) physician approval to participate.

Exclusion criteria include the following: (1) inability to read, write, or speak English; (2) inability to provide written informed consent; (3) history of type 1 diabetes or type 2 diabetes; (4) pregnant or planning to become pregnant during the intervention period; (5) breastfeeding; (6) use of anti-obesity medications; (7) participation in another weight loss program or intervention; (8) previous bariatric surgery or plan to have bariatric surgery during the study period; (9) use of glucose-lowering medications other than metformin; (10) blood disorders that require transfusion or phlebotomy, including anemia, hemoglobinopathies, or polycythemia; (11) adherence to a vegan or vegetarian diet; (12) adherence to a very low-carbohydrate diet; (13) difficulty chewing or swallowing; (14) inability to control foods that are purchased, prepared, or served; (15) untreated eating disorder or unstable serious mental illness (such as depression with suicidal ideation, bipolar or schizophrenia with psychosis); (16) abnormal baseline labs, including triglycerides ≥ 600 mg per deciliter (mg/dL), thyroid stimulating hormone (TSH) < 0.4 milli-international units per liter (mIU/L) or > 5.0 mIU/L, serum potassium < 3.6 mmol per liter (mmol/L) or > 5.2 mmol/L; (17) chronic kidney disease ≥ stage 4; (18) use of loop diuretic equivalent to furosemide 20 mg per day or greater; (19) warfarin use; (20) chronic oral corticosteroid use; and (21) any condition for which the study team deems participation to be unsafe or inappropriate.

### Who will take informed consent? {26a}

Study coordinators who are trained in research ethics, compliance, and consent procedures will obtain informed consent from all participants.

#### Consent for baseline blood draw

Potentially eligible participants who complete the online screening survey will receive an email from the study team that includes a link to a pdf blood draw consent form and a link to an orientation video explaining the study goals and procedures. Individuals will be asked to watch the video and then answer a brief questionnaire to assess their understanding of the study and to provide consent for a blood draw. Consent for the blood draw will be completed individually and online via REDCap [21]; potential participants are encouraged to contact the study team with any questions. Individuals that provide baseline blood draw consent will receive information by email regarding participating laboratory locations and hours.

#### Consent for full study participation

Individuals who complete the blood draw and have an HbA1c of 5.7 to 6.4%, triglycerides ≤ 600 mg/dL, TSH within normal range, and serum potassium within normal range will be informed of their study eligibility by email and invited to schedule a virtual visit with a trained study team member to complete study enrollment and consent processes. During this virtual visit, a study team member will review intervention procedures (e.g., randomization process), discuss expectation of participants (e.g., class attendance, completion of assessments), and answer any questions. Individuals that remain interested in study participation will be asked to provide written informed consent and will complete the consent online via REDCap [21] during the virtual visit with the study team. Participants will also complete a baseline survey via REDCap [21].

### Additional consent provisions for collection and use of participant data and biological specimens {26b}

Participants will complete informed consent for their first blood draw, which will confirm participant eligibility. Participants will have HbA1c, triglycerides, TSH, serum potassium, among other tests, at this time. Eligible participants will complete a main study consent, which will include consent for blood draws at months 4 and 12. Additionally, participants are required to consent to be recorded to participate in this trial within the main study consent form.

### Interventions

#### Explanation for choice of comparators {6b}

The NDPP is a low-fat, calorie restricted dietary intervention; it is the standard lifestyle change approach for T2DM prevention. Dietary carbohydrate restriction is effective for T2DM management, but less is known about its role in T2DM prevention. We will test the effectiveness of the NDPP versus a very low-carbohydrate adaption of the program.

#### Intervention description {11a}

All participants will be encouraged to engage in lifestyle changes to support weight loss and T2DM prevention. Both NDPP and VLC-NDPP groups will include 16 core sessions over 4 months followed by 8 monthly sessions. All sessions will last an hour and will be held on Zoom, in groups of approximately 15–18 participants. Sessions will be delivered online by coaches from the National Kidney Foundation of Michigan (NKFM), our local leader in the delivery of CDC-recognized DPPs [22], and our community partner in prior pilot studies [17, 23]. Session topics relate to dietary change, meal planning, grocery shopping, increasing physical activity, managing stress, and supporting self-efficacy for initiating and sustaining lifestyle change. The VLC-NDPP differs from the NDPP primarily in terms of its dietary advice; the content of the non-dietary sessions is minimally altered. Participants in both groups will be asked to self-weigh at least once weekly, maintain food logs, and track minutes of physical activity each week; these data will be collected via online survey prior to each session and shared with the coach. The coach will discuss any issues during group sessions and may contact individual participants, as needed.

#### National Diabetes Prevention Program (NDPP)

Participants will receive lifestyle change recommendations in accordance with the CDC’s NDPP, which teaches individuals to follow a low-fat, calorie-restricted diet and to engage in at least 150 min of moderate-intensity physical activity per week. With regards to dietary advice, participants are taught that fat contains more calories per gram than protein or carbohydrate and a reduction in dietary fat thus potentiates weight loss. Participants are encouraged to eat whole grains, vegetables, fruit, dairy, and lean protein throughout the day with the following explanation: “Spread your calories out through the day. Doing so helps keep you from getting too hungry and losing control. Eat 3 meals each day and 1 or 2 healthy snacks.” The NDPP includes handouts for participants and a detailed guide for coaches [24].

#### Very low-carbohydrate adaptation of the NDPP (VLC-NDPP)

Participants will receive lifestyle change recommendations in accordance with our adapted curriculum, which replaces low-fat dietary recommendations with very low-carbohydrate dietary recommendations. Program topics related to physical activity or behavioral change techniques were minimally altered. Table 1 summarizes the VLC-NDPP session topics and the degree to which components, such as diet, physical activity, and behavior change strategies were modified from the NDPP.

**Table 1:** Very low-carbohydrate-national diabetes prevention program topics and summary of changes made to the Center for Disease Control and Prevention’s National Diabetes Prevention Program

General topics and timing	Changes from standard NDPP	Session titles
Introduction (month 1)	Adds low-carbohydrate meal plan goals	• Welcome to the DPP
Nutrition (months 1–12)	Completely altered; now teaches how to follow a very low-carbohydrate meal plan	• Be a Carbohydrate Detective • What to Eat • Cooking and Shopping • Four Keys to Eating Out • Welcome to the Post-Core Phase • Fat: Saturated, Unsaturated, and Trans Fats • Handling Holidays, Vacations, and Special Events • Grocery Store Information • Revisiting Recipes and Cooking
Physical activity (months 1–12)	Adds strategies for being physically active when following a very low-carbohydrate meal plan	• Move Those Muscles • Being Active: A Way of Life • Jump Start Your Activity Plan • Staying on Top of Physical • Activity • Being Physically Active Together
Behavior change strategies (months 1–12)	Slightly adapted to include very low-carbohydrate food recommendations	• Challenges and Support • Take Charge of What’s Around You • Problem Solving • Talk Back to Negative Thoughts • The Slippery Slope of Lifestyle Change • Make Social Cues Work for You • You Can Manage Stress • Ways to Stay Motivated • Stress and Time Management • Long-Term Maintenance and Looking Forward

The VLC-NDPP teaches participants to reduce dietary carbohydrates to 20–35 g of non-fiber carbohydrates per day. Participants will be advised to reduce their carbohydrate intake back to their prior tolerated level if their weight increases. Participants will be encouraged to eat when they are hungry and stop when they are full, to keep their protein levels similar to baseline, presuming they are meeting the recommended dietary requirement [25], and to derive their remaining calories from fat. In general, participants are taught to avoid foods such as potatoes, rice, pasta, bread, donuts, and sugar-sweetened beverages; they are instructed to consume foods such as meat, fish, tofu, tempeh, full-fat dairy, eggs, fats, nuts, seeds, berries, and leafy or other low-carbohydrate vegetables. We provide information about low-carbohydrate versions of foods, such as eggplant-based lasagna, spiralized zucchini pasta, and cauliflower rice.

After the program’s core phase, if participants have reached their weight loss goal and desire to liberalize their carbohydrate intake, they are taught to gradually increase their carbohydrate consumption by adding no more than 5 net grams of carbohydrates to their daily goal per week. For example, an individual consuming 35 g of non-fiber carbohydrates per day may increase to 40 g of non-fiber carbohydrates per day and continue that goal for a minimum of 1 week before making additional changes.

### Training coaches

All coaches will receive standard Lifestyle Coach Training according CDC guidelines [26] to ensure consistency in the skills necessary to run group sessions. Both the NDPP and VLC-NDPP curricula include handouts for participants, as well as a detailed guide for coaches. VLC-NDPP coaches will receive additional training by study team members to ensure adequate content knowledge regarding dietary carbohydrate restriction. Following the training period, VLC-NDPP coaches will complete a knowledge assessment quiz. Individuals who score low on the quiz will be required to repeat training.

### Ensuring intervention fidelity

All sessions will be recorded. We will randomly select 20% of sessions, stratified by intervention phase (either core phase or maintenance phase), to assess curriculum fidelity. Study staff will review recordings of the group sessions and assess whether each class meets content objectives, including the extent to which the coaches (a) covered curriculum content, (b) maintained appropriate control over the pacing of the session, (c) troubleshot individual participant challenges effectively, (d) conveyed enthusiasm for the topics, (e) described the topics clearly, and (f) described the topics accurately. Coaches will receive feedback for classes with < 90% adherence to the protocol or low scores in any of these areas; additional training will be provided, as needed.

Coaches will report on every group session by completing an online survey immediately following the session. This survey will ask coaches to report any issues with participant adherence, adverse events, or side effects. Coaches will also be asked to self-evaluate their adherence to the curriculum content.

#### Criteria for discontinuing or modifying allocated interventions {11b}

Study physicians will use their clinical judgement to determine if participants should be removed from the trial due to the development of exclusionary diagnoses or addition of exclusionary medications (e.g., glucose-lowering medication other than metformin). We do not anticipate modifying or discontinuing modifying allocated interventions.

#### Strategies to improve adherence to interventions {11c}

The books “Calorie King” [27] and “Dana Carpender’s Keto Fat Gram Counter” [28] will be sent to NDPP and VLC-NDPP participants, respectively. All participants without access to a home scale will receive one by mail at the start of the trial.

#### Relevant concomitant care permitted or prohibited during the trial {11d}

Participants are encouraged to continue with their health care as normal throughout the trial. All blood test results will be sent to participants and their PCPs. Enrollment in other nutrition, weight, or diabetes-related trials or programs is prohibited. It is expected that some participants from either group may advance from prediabetes to T2DM during the study period. If PCPs decide to start their patient on glucose-lowering medications, those participants will be allowed to remain in the trial, except for sodium-glucose cotransporter-2 (SGLT-2) inhibitors. Participants who start an SGLT-2 inhibitor will be removed from the trial due to concerns for increased risk of euglycemic diabetic ketoacidosis when used with very low-carbohydrate diets.

#### Provisions for post-trial care {30}

We do not plan for any post-trial care. Any adverse events, reactions, or symptoms reported by participants during the trial will be addressed immediately. We do not anticipate the need for post-trial care or follow-up.

### Outcomes {12}

#### Primary outcome measure

##### Change in glycemic control

HbA1c is the most widely accepted measure of overall glycemic control in clinical care and predicts the risk of microvascular diabetes complications in people with type 2 diabetes [29]. HbA1c levels will be measured at 0, 4, and 12 months, and the change in HbA1c from baseline to 12 months will be the primary study outcome.

#### Secondary outcome measures

##### Mean change in body weight

Participants’ body weight will be measured using a calibrated scale at 0 and 12 months. Participants will also be advised to self-weigh at least once weekly using a home scale (provided by the study team, if necessary). Participants will report home weight data to their coach through a weekly online survey prior to each session. We will calculate average body weight change at 4 and 12 months compared to baseline.

##### Mean percent body weight loss

We will calculate percent body weight loss (100 − (weight at baseline/weight at 4 or 12 months multiplied by 100)).

##### Percentage of participants who achieve ≥ 5% and ≥ 10% body weight loss

We will calculate the percentage of participants who achieve ≥ 5% and ≥ 10% body weight loss at 4 and 12 months by dividing the number of participants per treatment arm who achieve these weight targets by the total number of participants in treatment arm and multiplying by 100.

##### Change in glycemic variability

Glycemic variability contributes to vascular damage [30], and intraday blood glucose variability is greater in people with prediabetes compared to people with normal glycemic levels [31]. We will place an Abbott Libre Pro continuous glucose monitoring (CGM) device on a participant’s upper arm at 0 and 12 months. Participants will wear each sensor for 14 days. This type of CGM records participants’ glucose levels in the interstitial fluid by a glucose oxidase method every 15 min; the sensor is blinded, and participants will not receive feedback. The research team will then download sensor data at the end of the measurement period. Following previous research, we will assess the glucose variability and the proportion of time spent in the euglycemic (3–7.8 mmol/l) and hyperglycemic (≥ 11.1 mmol/l for at least 15 min) states, following previous standards for interstitial glucose concentrations [32, 33].

##### Change in serum insulin and insulin resistance

Homeostatic Model Assessment-Insulin Resistance (HOMA-IR) is a widely used method of estimating insulin resistance based on fasting insulin and glucose levels [34]. HOMA-IR will be calculated at 0, 4, and 12 months and mean change from baseline to 4 and 12 months will be determined.

##### Change in serum lipids

Understanding how a carbohydrate-reduced diet affects lipids for those with prediabetes may help assess the likely impact of the diet on macrovascular complications. We will assess lipids using NMR Lipoprofile® by LabCorp [35].

##### Change in inflammatory markers

High-sensitivity CRP will be measured with nephelometric methods utilizing latex particles coated with CRP monoclonal antibodies and standardized against a CRP reference preparation at 0, 4, and 12 months.

#### Exploratory outcomes

##### Class attendance

We will report mean session attendance. Additionally, in accordance with CDC Diabetes Prevention Program Recognition Standards, we will report the number of participants that completed at least 8 sessions during the program’s core phase and who remained engaged in the program for at least 9 months [36].

##### Dietary adherence

We will assess dietary adherence with one unannounced 24-h dietary recall at 0, 4, and 12 months, which allows us to measure absolute and percent of calories of each macronutrient. For participants in the very low-carbohydrate group, blood ketone levels are a biomarker to help assess whether target levels of carbohydrate restriction have been achieved. We will assess fasting β-hydroxybutyrate at 0, 4, and 12 months for all participants.

##### Rate of conversion to T2DM

We will calculate the percentage of participants that progress from prediabetes to T2DM at 4 and 12 months, as determined by HbA1c level of ≥ 6.5%.

##### Initiation of anti-hyperglycemic agents

We will calculate the percentage of participants in each arm that initiate use of any anti-hyperglycemic medication during the study period.

##### Change in survey measures

We will evaluate change in survey measures, like self-reported symptoms and quality of life, from baseline to 4 and 12 months.

#### Qualitative data collection

##### Participants’ experiences in the intervention

We will conduct semi-structured interviews with a total of 68 participants; participants will have the opportunity to complete interviews at either 4 or 12 months after baseline. During these interviews, we will explore participants’ experiences during the intervention, including perceived barriers to and facilitators of dietary change and their ability to maintain these changes over time. We will purposively sample individuals with varied weight change and HbA1c outcomes. We will maximize sample variation by inviting participants with differences in gender, age, and race/ethnicity to complete interviews.

#### Participant timeline {13}

The participant timeline is shown in Table 2.

**Table 2:**
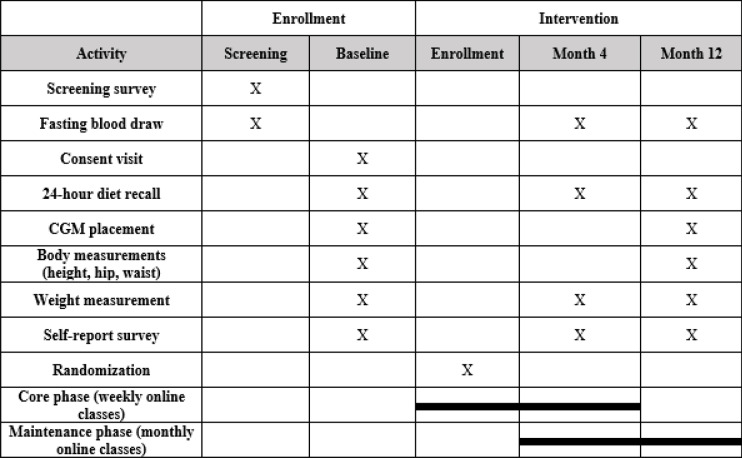
Participant timeline

#### Sample size {14}

To estimate the number of subjects to recruit, we note that the DPP showed an approximate 0.1% decrease in HbA1c with a standard deviation of the difference approximately equal to 0.1% [3]. This decrease in HbA1c was deemed clinically significant because it led to a clear reduction in type 2 diabetes incidence compared to control. Two studies of VLCDs in adults with prediabetes had a standard deviation for the difference approximately equal to 0.1%, similar to the DPP [37, 38]. Thus, we used 0.1% as the standard deviation in our sample size calculation. We used an alpha level of 0.05. Finally, although this is a superiority trial, the potential of non-inferiority is of clinical value; thus, we used a power of 95%. To be conservative, and because the low ICC (cited above) causes minimal increase in variability due to clustering, we consider a test of the difference between the two groups at 12 months. Our proposed hierarchical linear model that pools the repeated measurements will enable us to maintain high power while adjusting for important covariates like sex and examining the effect of treatment phase. The sample size needed to detect a 0.1% decrease in HbA1c with a standard deviation of 0.1% with alpha of 0.05 and power of 0.95 using a two-sample *t*-test is 105 participants per group. Our ongoing and published trials of diet and lifestyle programs with adults with prediabetes have had a retention rate of 70% or better [17]. Assuming a similar rate of 70% retention, the number of participants we need to enroll is 150 per group.

#### Recruitment {15}

Based on study inclusion criteria, we will use DataDirect [39], a self-serve tool that enables access to clinical data on more than 4 million unique patients within the University of Michigan Health System, to identify potentially eligible individuals. Potentially eligible individuals will be sent a letter with information about the study by postal mail or e-mail. The letter will include a web address to a website with trial information, study team contact information, and a link to an online screening survey. The screening survey will be used to obtain sociodemographic characteristics, HbA1c, weight, height, and medication use. If we are not able to meet our recruitment target with this initial strategy, we may recruit by (1) direct telephone outreach; (2) referral by primary care providers; and/or (3) posted flyers and advertisements on UMHealthResearch.org [40] or social media platforms, such as Facebook™.

### Assignment of interventions: allocation

#### Sequence generation {16a}

Individuals who meet study inclusion criteria, provide written informed consent, and complete baseline assessments will be randomized to one of two groups, standard or very low-carbohydrate version of the DPP, with a 1:1 ratio. The order will be created using block randomization procedures, with blocks randomly allocated to size 2, 4, or 8 and two strata: baseline HbA1c of 5.7–6.0% or 6.1–6.4% and sex of male or female. We will use the R package blockrand() with a random seed of 2345.

#### Concealment mechanism {16b}

A web-based tool will be used for blinded treatment allocation.

#### Implementation {16c}

A trained study team member will notify participants of their treatment assignment.

### Assignment of interventions: blinding

#### Who will be blinded {17a}

Although treatment condition will be apparent to participants and researchers, outcome assessment and data analyses will be blinded. Participants will be made aware of their treatment group assignment via email 1 week prior to the first session.

#### Procedure for unblinding if needed {17b}

Unblinding will not occur in this trial as only data analysts and outcome assessors will be blinded. Participants will not be blinded.

### Data collection and management

#### Plans for assessment and collection of outcomes {18a}

##### Pre-enrollment

The following laboratory testing will be obtained when individuals present for their screening (baseline) HbA1c: (1) lipids, (2) serum insulin, (3) fasting plasma glucose, (4) high-sensitivity C-reactive protein (hsCRP), (5) fasting β-hydroxybutyrate (ketones), (6) a comprehensive metabolic panel, and (7) TSH.

##### Baseline

Consented individuals will be asked to schedule an in-person appointment at Michigan Diabetes Research Center’s Clinical Research Unit to complete the following procedures: (1) height, hip, and waist measurements; (2) body weight measurement; and (3) continuous glucose monitor (CGM) placement. Participants will wear their CGM (which will not provide feedback to participants) for 2 weeks before mailing it back to the study team. Following consent and prior to the start of the intervention, participants will be contacted by phone for one unannounced 24-h dietary recall. Within the 2 weeks before classes begin, participants will be asked to complete an online survey; survey items will assess self-reported symptoms (using measures adapted from the literature [41]), quality of life [42], and medication use.

##### Four months

The following measures will be collected at 4 months: (1) HbA1c, (2) lipids, (3) serum insulin, (4) fasting plasma glucose, (5) hsCRP, (6) fasting β-hydroxybutyrate, (7) comprehensive metabolic panel, and (8) body weight as measured on home scale. Participants will be asked to complete an online survey (the same one they completed at baseline with added items to assess program satisfaction) and will complete one unannounced 24-h dietary recall.

##### Twelve months

The following laboratory measures will be collected at 12 months: (1) HbA1c, (2) lipids, (3) serum insulin, (4) fasting plasma glucose, (5) hsCRP, (6) fasting β-hydroxybutyrate, and (7) comprehensive metabolic panel. Participants will also complete an in-person appointment at Michigan Diabetes Research Center’s Clinical Research Unit for the following procedures: (1) height, hip, and waist measurements; (2) body weight measurement; and (3) CGM placement. Participants will wear their CGM (which will not provide feedback to participants) for 2 weeks before mailing it back to the study team. Participants will be asked to complete an online survey (the same one they completed at baseline with added items to assess program satisfaction) and will complete one unannounced 24-h dietary recall.

#### Plans to promote participant retention and complete follow-up {18b}

To encourage participant retention, participants will be paid $55 at 4 months and $75 at 12 months for completing study-related assessments at each time point. Participants will receive an additional $20 at baseline for returning their CGM on time. Additionally, individuals who participate in optional semi-structured interviews will receive $25.

#### Data management {19}

Study data will be collected and stored in REDCap [21] or University of Michigan’s HIPAA-compliant DropBox account [43]. All computers used by study staff will be encrypted. All blood drawn from participants will be analyzed and then destroyed by LabCorp. Laboratory test results will be shared with participants using secure email. Participants’ physicians may be contacted through the Electronic Health Record, HIPAA-compliant fax, or by phone. Participant data will be retained by the study team until analyses are complete. Data related to any identifying information will be destroyed once analyses are complete (approximately 3–6 years post-study). All data will be kept in encrypted storage.

#### Confidentiality {27}

We will use a crosswalk file to link participants to a unique study specific identifier. The identifier will be used, whenever possible, in place of identifiable participant information.

#### Plans for collection, laboratory evaluation, and storage of biological specimens for genetic or molecular analysis in this trial/future use {33}

Participants will have blood samples drawn during screening and at months 4 and 12. Participants will go to any LabCorp clinic location to have their blood drawn. All samples will be analyzed by LabCorp and destroyed after analysis.

### Statistical methods

#### Statistical methods for primary and secondary outcomes {20a}

##### Primary intent-to-treat analysis

The principal analysis will be intent-to-treat, with all observations included for all individuals based on initial group assignment, regardless of adherence to the treatment protocol. Our primary outcome is a continuous longitudinal outcome, so we will use hierarchical linear mixed effects models to assess differences over time. The final model will include fixed effects for linear and quadratic time-by-arm interaction terms and randomization and stratification variables. Covariance terms will be included for repeated measures over time. Because we are studying a group intervention, there may be effects that vary due to differences in group dynamics or teachers. To address this, we will include a cluster variable as a random effect to account for the lack of full independence of observations within the same group. Our previous trials’ intraclass correlation coefficient (ICC) for the clustering effect of coaches has been < 0.001, suggesting that the effect of individual dynamics of groups is minimal. We will investigate model diagnostics for the linear model. If the assumption of normal errors is not met, we will consider transformations, such as the log transform, to reduce skewness.

For secondary outcomes that are continuous, we will use linear mixed models that are analogous to the model described above. For secondary outcomes that are binary (e.g., percentage of participants who achieve weight loss thresholds), we will use an analogous general linear mixed effects model. All analyses will be conducted using either SAS or R statistical software.

#### Qualitative analysis

Interviews will be recorded and transcribed verbatim by a professional transcriptionist. At least two members of the study team will participate in inductive, thematic data analysis, which will involve the following: (1) reviewing transcripts; (2) developing codes; (3) applying codes to transcripts; (4) resolving coding differences through consensus conferences; (5) developing themes that interrelate codes; (6) reviewing and refining themes; and (7) describing themes, evidenced by participant quotes [44].

#### Integrated analysis

Mixed methods integration occurs when quantitative and qualitative data are combined to further clarify the research question [45]. We will integrate the data in several ways. Following thematic analysis, we will use the resulting codes and themes to create categorical variables of barriers and facilitators of dietary changes. This approach has been used in health services research to develop variables that can be tested in subsequent quantitative analyses [46, 47]. Using this approach, we will explore the association between variables derived from qualitative data and quantitative intervention outcomes. Second, we will create a visual joint display to co-present qualitative and quantitative outcomes and enhance our understanding of the study’s findings [48].

#### Interim analyses {21b}

As this is a low-risk intervention, there will be no formal stopping rules and no interim analyses will be performed.

#### Methods for additional analyses (i.e., subgroup analyses) {20b}

##### Secondary per-protocol analyses

As a secondary approach, we will also perform a per protocol analysis based on session attendance. The CDC Diabetes Prevention Recognition Program defines a program completer as “an eligible participant enrolled in an evaluation cohort who attended at least 8 sessions in months 1–6 and whose time from first session held by the cohort to last session attended by the participant is at least 9 months.” [49]. We will analyze our results based on CDC completer status and also by using session attendance as a continuous measure.

#### Methods in analysis to handle protocol non-adherence and any statistical methods to handle missing data {20c}

To investigate the impact of missing data on the analysis, we will examine the pattern of missingness in the data. We will compare the means among each observed pattern of missingness. If the means are similar, suggesting that the data are missing completely at random, then we will exclude missing values from the analysis. If the pattern of missingness suggests that the data are missing not at random, we will use multiple imputation to estimate the treatment effect while correctly modeling the variability in the data.

#### Plans to give access to the full protocol, participant level-data, and statistical code {31c}

The study’s full protocol, datasets analyzed, and statistical code will be available from the corresponding author on reasonable request and with the proper regulatory permissions.

### Oversight and monitoring

#### Composition of the coordinating center and trial steering committee {5d}

University of Michigan is the coordinating center for this trial. Dr. Saslow is the principal investigator. She will meet with co-investigators Drs. DeJonckheere, Griauzde, Isaman, Richardson, and Yancy at least once monthly.

#### Composition of the data monitoring committee, its role and reporting structure {21a}

The Data Safety and Monitoring Board (DSBM) is independent from the sponsor, has not worked with the study team, and does not have competing interests. The DSBM will meet every 6 months to review study procedures and adverse events, should they occur.

#### Adverse event reporting and harms {22}

The team will ensure continuous and close monitoring of participant safety. The team will report to the DSMB (Data and Safety Monitoring Board). Study progress and safety will be reviewed weekly by the principal investigator (PI) and core study team. Unexpected fatal or life-threatening adverse events (AEs) related to the intervention will be reviewed by the study team as they occur and reported to the DSMB, IRB, and NIH Program Officer within 3 days of the study team becoming aware of the event. Other serious and unexpected AEs related to the intervention will be reported within 5 working days. Anticipated or unrelated serious adverse events (SAEs) will be handled in a less urgent manner but will be reported to the DSMB, IRB, and other oversight organizations in accordance with their requirements and will be reported to NIH on an annual basis. All other AEs documented during the trial will be reported to NIH on an annual basis by way of inclusion in the annual report and in the annual AE summary which will be provided to NIH and to the Independent Monitors. The DSMB Report will state that all AEs have been reviewed. Investigators will include the following information when reporting an adverse event, or any other incident, experience, or outcome as an unanticipated problem to the IRB: (1) appropriate identifying information for the research protocol, such as the title, investigator’s name, and the IRB project number; (2) a detailed description of the adverse event, incident, experience, or outcome; (3) an explanation of the basis for determining that the adverse event, incident, experience, or outcome represents an unanticipated problem; and (4) a description of any changes to the protocol or other corrective actions that have been taken or are proposed in response to the unanticipated problem. AEs will be followed for outcome information. Study personnel will follow-up with participants on a regular basis (frequency to be determined by the nature of the problem) until the problem has resolved or stabilized. These contacts may be made by the study physicians or personnel (including staff) and will be done via phone and/or email or text as the PI deems appropriate depending on the event and situation and considering participant preference as reasonable.

#### Frequency and plans for auditing trial conduct {23}

Twice yearly DSMB meetings will be held via teleconference. The DSMB will be able to recommend amendments to the protocol, changes in study procedures, changes to the data collection plan or study forms, or study termination due to safety or other issues. If a SAE is identified, the DSMB Chair will schedule a full meeting of the DSMB and review the results of the SAE report from the University of Michigan IRB prior to this meeting and to determine what changes if any are necessary and if the RCT should be stopped. The decisions of the IRB and the DSMB will assist the team with developing plans to implement this RCT in a manner that minimizes research-related risks to participants. Thus, the DSMB will meet regularly to review the study progress, review modifications, and monitor compliance with IRB rules, human subjects’ procedures, and IRB regulations. The principal investigator will provide oversight of all study procedures and quality assurance checks.

The project management team, including the study coordinator(s), PI, and other key personnel, will meet weekly to discuss trial progress, recruitment, participant concerns, etc. The full project team, including co-investigators, will meet monthly to discuss trial progress, recruitment, participant concerns, and to review trial protocols and procedures.

#### Plans for communicating important protocol amendments to relevant parties (e.g., trial participants, ethical committees) {25}

Should the DSMB make recommendations to amend the study protocol or terminate the study, these recommendations and planned responses will be forwarded to the NIH program officer within 10 working days. Should the protocol be amended because of data review, the IRB will be notified, and the amendment approved prior to study amendment implementation unless the protocol amendment must be implemented to protect the immediate safety of the study subjects. In such a case, the protocol amendment will be immediately implemented, and the IRB will be notified directly after protocol amendment implementation. Additionally, the protocol will be updated in Clinicaltrials.gov.

#### Dissemination plans {31a}

To ensure the outputs from this research inform practice and policy change, the following dissemination strategy has been developed:

- Dr. Saslow will share information about this study via timely registration, updates, and results reporting in ClinicalTrials.gov in accordance with NIH policy.
- The informed consent documents used for this study will include statements to inform participants that information about the trial will be posted in ClinicalTrials.gov.
- In addition, we will disseminate our findings at academic research conferences and peer-reviewed publications.
- We may work with University of Michigan’s Institute for Health Policy and Innovation’s public and government relations staff to disseminate our findings through the lay press, media, and to government stakeholders.
- Finally, and most importantly, this patient-centered proposal will make an active effort to report our findings back to patient groups.

## Discussion

In the USA, 88 million adults have prediabetes [50] and face an elevated lifetime risk of T2DM [51] and cardiovascular disease [52]. The CDC’s NDPP is an evidence-based program that aims to help individuals with prediabetes avoid health complications by achieving modest weight loss though diet and physical activity changes. However, most program participants do not achieve the program’s goal of ≥ 5% body weight loss. One opportunity to augment the NDPP’s effectiveness may be teach participants to follow a carbohydrate-restricted rather than a fat-restricted eating pattern. To our knowledge, this will be the first large-scale study testing the comparative effectiveness of the NDPP versus a very low-carbohydrate adaptation of the program’s dietary advice.

The VLC-NDPP capitalizes on the popularity of carbohydrate-restricted eating patterns [53, 54] and is also substantiated by nutrition science advances. Specifically, there is growing recognition of the central role of hyperinsulinemia in the pathogenesis of obesity [14, 55] and chronic diseases such as T2DM [56–58]. This contrasts with prior literature suggesting that hyperinsulinemia occurs secondary to weight gain and insulin resistance [59]. Insulin is an anabolic hormone that inhibits lipolysis and promotes lipogenesis [60], and dietary carbohydrate—as opposed to dietary protein or fat—is the strongest driver of post-prandial glycemia and resultant insulin secretion [61]. Thus, dietary carbohydrate restriction reduces serum glucose and insulin levels and enables a reduction in body weight via lipolysis [62]. The potential benefits of low- and very low-carbohydrate diets are well-established among patients with T2DM [11, 12, 16], and results from this trial will contribute to emerging data regarding the role of carbohydrate restriction among patients with prediabetes [11, 63, 64].

We hypothesize that the VLC-NDPP will demonstrate greater glycemic and weight loss effectiveness than the NDPP. Pending favorable clinical trial results, we plan to submit the VLC-DPP curriculum to the CDC for approval, as it adheres to the organization’s curriculum requirements, including emphasis on preventing T2DM through sustainable lifestyle change and achievement of modest weight loss [65]. This could enhance the population health impact of our work, as organizations that provide programs for T2DM prevention would have access to an alternative CDC-approved curriculum. Notably, we acknowledge the limitations of any “one-size-fits-all” approach to lifestyle change and recognize that average treatment effects across all dietary interventions should be interpreted in the context of wide individual-level variation in outcomes [66, 67]. Such treatment effect heterogeneity reflects, in part, differences in individuals’ preferences and needs. Accordingly, the VLC-NDPP may emerge as an evidence-based option for T2DM prevention that can be employed as part of a personalized, preference-sensitive approach to lifestyle change rather than a replacement for the NDPP.

## Limitations

This study has several potential limitations. First, we will plan to primarily recruit patients from a single academic medical center, which may limit the study’s generalizability. However, the medical center serves patients throughout Southeast Michigan with racial/ethnic characteristics comparable to 2016 U.S. Census Data estimates for the state of Michigan (i.e., 80% White, 14% African American, 5% Latino, 3% Asian). We may also use alternative recruitment strategies, such as social media, which will enable recruitment from a broader geographic area. Moreover, the online nature of the intervention will similarly facilitate geographic diversity, though participants will need to be on site on two occasions for in-person assessments. Second, this trial is not powered to detect incident T2DM among NDPP vs. VLC-NDPP participants. However, change in HbA1c is the study’s primary outcome, which will advance current knowledge about glycemic change among participants in community-based interventions to T2DM.

## Conclusion

This trial aims to provide critical data to support the use of an alternative curriculum for T2DM prevention. If the VLC-NDPP intervention shows promise, this trial would be a precursor to a multi-site trial with incidence of type 2 diabetes as the primary outcome.

## Trial status

Recruitment for this study began in March 2022 and is expected to be completed by July 2025.

## Data Availability

Data is not anticipated to be publicly available. Study materials are available from authors on request.

## Abbreviations

AEs: Adverse events
CDC: Centers for Disease Control and Prevention
CGM: Continuous glucose monitor
DPP: Diabetes Prevention Program
DPRP: Diabetes Prevention Recognition Program
DSBM: Data Safety and Monitoring Board
HbA1c: Hemoglobin A1c
hsCRP: High-sensitivity C-reactive protein
HOMA-IR: Homeostatic Model Assessment-Insulin Resistance
ICC: Intraclass correlation coefficient
NDPP: National Diabetes Prevention Program
NKFM: National Kidney Foundation of Michigan
PCP: Primary care provider
SAEs: Serious adverse events
SGLT-2 inhibitors: Sodium-glucose cotransporter-2 inhibitors
T2DM: Type 2 diabetes mellitus
VLCD: Very low-carbohydrate diet
VLC-DPP: Very low-carbohydrate National Diabetes Prevention Program
